# Quality of emergency oncological surgery: time for advanced oncological life support

**DOI:** 10.1590/1806-9282.2024S109

**Published:** 2024-06-07

**Authors:** Fábio de Oliveira Ferreira, Tibério Moura de Andrade Lima, Edivaldo Massazo Utiyama, Alexandre Ferreira Oliveira, Luiz Carlos Von Bahten, Heber Salvador de Castro Ribeiro

**Affiliations:** 1 Universidade de São Paulo, Clinical Hospital, Cancer Institute, Faculty of Medicine – São Paulo (SP), Brazil.; 2 Universidade de São Paulo, Clinical Hospital, Cancer Institute, Faculty of Medicine, Surgical Clinic Division – São Paulo (SP), Brazil.; 3 Universidade de São Paulo, Faculty of Medicine, Department of Surgery, Surgical Clinic Division – São Paulo (SP), Brazil.; 4 Federal University of Juiz de Fora, School of Medicine, Faculty of Medicine, Departament of Surgery – Juiz de Fora (MG), Brazil.; 5 Pontifical Catholic University of Paraná, Clinical Surgery – Curitiba (PR), Brazil.; 6 Federal University of Paraná, Department of Surgery – Curitiba (PR), Brazil.; 7 Pontifical Catholic University of Paraná, Cajuru University Hospital, Department of Surgery – Curitiba (PR), Brazil.; 8 A.C.Camargo Cancer Center, Department of Surgical Oncology – São Paulo (SP), Brazil.

**Keywords:** Cancer care facility, Healthcare quality assessment, Healthcare quality indicators, Medical emergency services, Surgical oncology, Operative procedures

## Abstract

In the emergency care of cancer patients, in addition to cancer-related factors, two aspects influence the outcome: (1) where the patient is treated and (2) who will perform the surgery. In Brazil, a significant proportion of patients with surgical oncological emergencies will be operated on in general hospitals by surgeons without training in oncological surgery.

**OBJECTIVE:**

The objective was to discuss quality indicators and propose the creation of an urgent oncological surgery advanced life support course.

**METHODS:**

Review of articles on the topic.

**RESULTS:**

Generally, nonelective resections are associated with higher rates of morbidity and mortality, as well as lower rates of cancer-specific survival. In comparison to elective procedures, the reduced number of harvested lymph nodes and the higher rate of positive margins suggest a compromised degree of radicality in the emergency scenario.

**CONCLUSION:**

Among modifiable factors is the training of the emergency surgeon. Enhancing the practice of oncological surgery in emergency settings constitutes a formidable undertaking that entails collaboration across various medical specialties and warrants endorsement and support from medical societies and educational institutions. It is time to establish a national registry encompassing oncological emergencies, develop quality indicators tailored to the national context, and foster the establishment of specialized training programs aimed at enhancing the proficiency of physicians serving in emergency services catering to cancer patients.

## INTRODUCTION

Cancer currently stands as the second-leading cause of death worldwide. In parallel with the estimated 50% increase in incidence and 62% rise in mortality by 2040, the care of oncology patients has become increasingly complex^1^. In many countries, due to the lack of large-scale screening programs, a significant portion of patients will receive a cancer diagnosis following an acute event in an emergency setting. In this scenario, apart from the aspects related to the underlying disease, two factors influence the outcome: (1) where the patient is treated and (2) who will perform the surgery. In Brazil, most cancer patients seeking emergency care undergo surgery in general hospitals, with access to specialized hospitals limited to patients with previously confirmed diagnoses. Consequently, surgical oncology emergencies are often managed by nonspecialized surgeons. Adding to this challenge is the difficulty of accessing tertiary and quaternary hospital emergency services, which consequently leads to emergency surgeries being performed predominantly in smaller hospitals, where the on-call surgeon, quite often, is in the early stages of their career.

Certainly, emergencies introduce conditions that impact clinical outcomes and the prognosis of oncology patients; however, it is essential to analyze the potential factors that justify the poorer results. Are the technical principles of oncological surgery being adhered to? Do patients exhibit distinct sociodemographic and clinical characteristics? Is there a lack of hospital infrastructure and/or professional expertise to address surgical oncology emergencies? It is intuitive to think that the ideal professional to attend to cancer patients seeking emergency care would be someone with experience in emergency surgery and a deep understanding of the fundamentals and principles of oncological surgery. However, it is foreseeable that the number of surgeons with this profile will be limited to meet demand.

Working to enhance the practice of oncological surgery in emergency conditions is a formidable task that involves various specialties and deserves support and encouragement from medical societies and educational institutions. In this paper, we analyze how to evaluate the quality of oncological surgery in an emergency setting and discuss proposals for improving outcomes.

## QUALITY INDICATORS IN ONCOLOGICAL SURGERY

Quality in healthcare is defined as safe, effective, patient-centered, timely, efficient, and equitable medical care. Quality reflects how medical care increases the likelihood of desired outcomes and reduces the likelihood of undesired outcomes^1^. However, measuring the quality of oncological surgery is not an easy task. Some challenges include the lack of a specific definition and the constant change in quality indicators (QIs) due to advances in oncological treatment^2^. QIs are standardized measures used to quantify and track the quality of care. QIs enable the assessment of aspects related to structure (e.g., number of board-certified physicians, nurse-to-bed ratio, hospital size), process (diagnostic, e.g., completeness of staging, adequacy of pathological specimen examination; treatment, e.g., completeness of neoadjuvant therapy, an adequate number of harvested lymph nodes), and outcome (morbidity, mortality, survival, quality of life, preservation of organ function)^3^. Other indicators are based on rates (e.g., anastomotic leak rate) and sentinel events, which are individual undesirable events requiring further investigation.

The collection and analysis of QI that illustrate the degree of compliance with predefined standards define the assessment of quality. Particularly in an emergency setting, the issue is that data is not always of high quality or readily available. Some services use administrative data, including postoperative mortality rate, postoperative hospital transfer rate, and hospital length of stay, but there is a lack of planning for prospective data collection. In practice, there is a need to establish management programs with a predefined QI adjusted to the national context so that issues can be identified and solutions discussed. Among patients seeking emergency services in Brazilian general hospitals, how many are oncology cases? What are the most common conditions? How many cases qualify as surgical emergencies? What are the rates of operative morbidity and mortality? What are the infection rates? How many patients have the minimum number of sampled lymph nodes? What is the percentage of non-oncological resections, and why? Among surgeons working in emergency settings, how many have supplementary training in surgical oncology? What is the volume-outcome relationship? Questions like these serve as QI and need to be part of management strategies so that we can conduct a proper analysis of reality and take appropriate actions.

## ACCREDITATION, VOLUME-OUTCOME RELATIONSHIP, AND SPECIALIZATION

Participation in accreditation programs demonstrates a commitment to quality objectives and goals; however, in an emergency setting, adherence to established standards is more vulnerable. In practice, due to demand, many patients are treated at low-volume centers by early-career surgeons with no basic training in surgical oncology, which increases the likelihood of undesirable outcomes.

A positive relationship between higher volume and better outcomes (volume-outcome relationship) has consistently been demonstrated in complex cases in surgical oncology. To understand the observed gain in the volume-outcome relationship, we must examine which specific attributes justify better results in high-volume centers, i.e., what are the routines, guidelines, and practices applied to each oncological condition, including emergencies. However, how can we replicate these results nationally given the heterogeneity in quality-of-service delivery? If achieving results in high-volume oncological centers is challenging to replicate in smaller centers for elective cases, certainly the emergency condition adds additional complexity when considering the variety of clinical situations and the greater difficulty in standardizing practices. Drawing a parallel with the care of traumatic emergencies, the collaborative efforts of medical societies, educational institutions, and private initiatives in organizing and disseminating guidelines for the management of trauma victims were notable, resulting in more balanced outcomes despite the significant disparities in emergency care conditions nationwide. In the case of emergency oncological surgery, besides the lack of minimal standardization, there is no proper notification or recording, rendering the assessment of the volume-outcome relationship imprecise or unavailable.

In any surgical field, specialization adds knowledge that translates into improved outcomes, which also applies to emergency and oncological surgery. However, unlike what happened with trauma surgery, which was organized to provide specialized training in the care of polytrauma patients, oncological surgery has not yet directly addressed this issue. Evidence of this is the lack of any formal curriculum requirement in surgical oncology for the hiring of surgeons working in emergency settings.

These issues pave the way for discussing the training of surgeons working in emergency services, as the 3 years of general surgery residency do not include official systematic rotations in surgical oncology. Therefore, one cannot demand enhanced technical knowledge from newly graduated surgeons who did not choose a specialization, many of whom will often be at the forefront of surgical oncological emergencies. This fact highlights the need for attention to training programs that provide qualifications for work in emergency surgery services, including courses such as Advanced Trauma Life Support (ATLS), Advanced Cardiac Life Support (ACLS), Pediatric Advanced Life Support (PALS), and Basic Life Support (BLS), among others, which have become formal requirements for employment in some hospitals seeking quality certification, including the need for periodic updates. It is time to create a certification program aimed at improving the training of surgeons working in emergency services and caring for cancer patients.

## SPECIFICITIES OF ONCOLOGICAL SURGERY IN THE EMERGENCY SETTING

In the emergency setting, there is often not a suitable condition for a comprehensive diagnostic investigation, either due to technical impossibility stemming from clinical limitations or due to a lack of infrastructure. Many emergency services lack the necessary imaging resources for minimal staging, and only a few have the privilege of having high-quality computed tomography or 24-h endoscopy services available.

The concept of the importance of multidisciplinarity in oncology, based on the results produced by the cooperative work approach adopted in major world reference centers, has become synonymous with good medical practice. However, in an emergency setting, how can multidisciplinary discussion be promoted? Depending on the workplace and the urgency of the situation, the multidisciplinary team will often be limited to the on-call surgical team. While complex cases are discussed in "tumor boards" in elective situations, in emergencies, solitary decision-making becomes common, where oncological complexity is compounded by unfavorable clinical conditions and a lack of adequate preparation time. In a population with unique clinical characteristics, including a significant fraction of elderly patients with comorbidities and advanced-stage cancer, this is a challenging reality to transform.

The knowledge and skill of the surgeon are crucial in oncological surgery; however, assessing a surgeon's actions in an emergency setting in terms of quality metrics is not an easy task. Small acts that occur within the operating room have the potential to impact the outcome and may not always be transparently recorded. In most cases, when analyzing a description of an oncological procedure in an emergency setting, it is inadequate. There is a need to raise awareness among emergency surgeons about the importance of proper documentation containing a description of findings and surgical staging (sTNM), explicitly stating findings related to the primary tumor, lymph node status, and the presence or absence of metastases. In addition to the technical details that confirm or deny adherence to oncological principles, it must be clear whether the operation was curative or palliative. This topic warrants collective effort from medical entities involved in emergency surgery and surgical oncology to establish national standardizations and minimal requirements. Only then can we identify the regions and services that require more investment in training and capacity-building, with the ultimate beneficiary being the cancer patient.

From this analysis, it is evident that the equation for assessing the quality of emergency oncological surgery is not resolved. The rationale is to maintain adherence to technical principles; however, the emergency condition adds obstacles.

## QUALITY OF EMERGENCY ONCOLOGICAL SURGERY: COLORECTAL CANCER AS AN ANALYTICAL MODEL

Approximately 15–40% of colorectal cancer (CRC) patients seek emergency services due to complications arising from undiagnosed disease, 8–40% due to obstruction, and 3–10% with intestinal perforation^4–6^. Both the demand for emergency services and the availability of data for comparison with elective surgery conditions make CRC a suitable model to answer the question about the quality of oncological surgery in the emergency setting. Are CRC patients well operated on in the emergency setting? Fundamental aspects involve the quality of diagnosis and staging, choice of surgical approach, selection of the type of operation concerning the extent of resection, primary anastomosis or external diversion, the number of sampled lymph nodes, the status of surgical margins, as well as clinical and oncological short- and long-term outcome indicators: morbidity rates, mortality, disease-free survival (DFS), and overall survival (OS). Additionally, the impact of surgeon training, the volume-outcome relationship, and the sociodemographic characteristics of the CRC patient population seeking emergency care need to be analyzed.

A literature review highlights differences in management and outcomes when comparing elective and nonelective surgery scenarios. CRC patients undergoing emergency surgery are less likely to undergo staging examinations and are less likely to be approached laparoscopically^7^. The type of operation is also influenced by the emergency setting, with a significantly higher number of Hartmann's procedures, stoma creation, and segmental resections at the expense of classic colectomies with primary anastomoses^8,9^. In emergencies, there is a higher likelihood of positive margins, a lower number of sampled lymph nodes^10,11^, and a higher anastomotic dehiscence rate^9^.

Morbidity and mortality rates are consistently higher in the emergency setting, as are hospitalization stays and rates of readmissions and reoperations^7,10,11^. In most studies, 5-year DFS and OS rates are significantly lower in the emergency group^8,10^. It is a fact that cases in emergencies are more advanced^12^, and there is a high proportion of elderly patients (including octogenarians) with clinical and socioeconomic problems^11,13^. Some authors even suggest that the worse outcomes are a consequence of patient profiles and clinical circumstances rather than the emergency itself^14^.

In emergencies, there is a lower likelihood that the patient will be operated on by a surgeon with a specialization in oncological or colorectal surgery, and resections are more likely to occur in community hospitals and low-volume centers^10^. In the analysis by Patel et al.^15^, elective surgeries were performed by colorectal surgeons (37%), oncological surgeons (10%), and general surgeons (53%); in emergencies, the proportion was: colorectal surgeons (19%), oncological surgeons (10%), and general surgeons (70%). The data reinforce the observation that the majority of CRC cases are operated on by general surgeons, both electively and in an emergency setting, emphasizing the importance of training emergency surgeons in oncological surgery.

This analysis demonstrates that nonelective resection for CRC is associated with a higher likelihood of short-term adverse outcomes, including higher rates of postoperative complications, mortality, stoma creation, admission to intensive care units, as well as poorer DFS and OS rates^16–18^. In this context, among the potentially modifiable factors are the training of emergency surgeons to handle CRC cases and the referral of more complex cases to higher-volume centers^19^.

## WHAT CAN BE DONE?

One of the suggestions to make the quality management process feasible is applying the plan-do-check-act (PDCA) cycle^1^. The planning phase could involve a joint effort by entities such as the Brazilian Society of Oncological Surgery (SBCO) and the Brazilian College of Surgeons (CBC) to establish QIs of recognized importance in emergency oncological surgery. Once the QIs are defined and a data recording platform is created, teaching and educational strategies focused on surgical skills training will be established in partnership with these entities. The next steps would involve verifying the results of the initial measures and directing actions to strengthen the training of surgeons in managing oncological emergencies, starting in regions and hospitals where the indicators point to the greatest problems.

The success of quality improvement initiatives depends on the validity of the collected data and the reliability of the selected measures. Therefore, quality improvement programs that provide high-quality data are necessary. In the United States, the American College of Surgeons National Surgical Quality Improvement Program (ACS NSQIP)^20^ and the Agency for Healthcare Research and Quality Patient Safety Indicators (AHRQ-PSIs) have been established as tools for measuring surgical quality^21^. The ACS NSQIP is a nationally validated, risk-adjusted outcomes-based program for quality measurement and improvement with more than 600 participating hospitals. There is solid evidence that enrollment in quality improvement programs helps hospitals improve surgical quality over time. An analysis of 118 hospitals from the private sector participating in the ACS NSQIP showed that 66% of the hospitals reduced risk-adjusted mortality and 82% had a decreased complication rate^20^. The American Society of Clinical Oncology (ASCO) has established an infrastructure with more than 70 specific quality measures for cancer treatment^22^. The European Organization for Research and Treatment of Cancer (EORTC) initiated the first quality assurance projects in the 1980s^23^. In 2007, EURECCA (EUropean REgistry of Cancer CAre or EURopEan CanCer Audit) was created with the aim of improving the quality of cancer patient care^24^. In addition to programs focused on quality assessment, numerous Operative Standards Manuals for Cancer Surgery have been created under the supervision of the American College of Surgeons (ACS)^25^. This is an idea that could be developed by the SBCO in partnership with the CBC: the creation of manuals with guidelines for managing the most common oncological emergencies.

The quality of oncological surgical care is a priority in various healthcare systems, with ongoing efforts to reliably measure surgical outcomes. Particularly in the emergency setting, knowledge of oncological surgical principles can assist in decision-making and, above all, positively influence the outcomes of cancer patients. It is time to establish a national registry that encompasses oncological emergencies; it is time to promote the creation of the "Advanced Oncological Life Support" ("AOLS") with the aim of training surgeons working in emergency services and caring for cancer patients.
